# A new method with commonly available devices for treating buried bumper syndrome

**DOI:** 10.1055/a-2439-3681

**Published:** 2024-11-13

**Authors:** Xiaoxiong Guo, Miao Liu, Canmei Zhong, Sihan Zhang, Liying Lin, Mingkai Zhuang, Fenglin Chen

**Affiliations:** 1 117890 Department of Gastroenterology, Fujian Medical University Union Hospital, Fuzhou, China

Buried bumper syndrome (BBS) is a rare yet significant complication following percutaneous endoscopic gastrostomy (PEG) that necessitates prompt intervention following diagnosis 1 2 . There are many methods available for treating BBS, each requiring distinct devices, some of which may need to be specifically dedicated, along with complex endoscopic techniques to guarantee effective treatment 3 . Therefore, we explored the use of commonly available devices, namely hot biopsy forceps and a polypectomy snare, to successfully and efficiently manage a case of BBS ( Video 1 ).

Release of buried bumper using hot biopsy forceps and polypectomy snare, and replacement of percutaneous endoscopic gastrostomy device and jejunal tube.Video 1

A 63-year-old patient with a history of long-term enteral nutrition via a PEG–jejunum (PEG-J) tube was admitted to our hospital with symptoms of redness and swelling around the insertion site, as well as difficulty in pushing the PEG tube into the stomach. Following an endoscopic examination, the patient was diagnosed with complete BBS.

We used the position of the jejunal tube to locate the center of the buried bumper ( Fig. 1 **a** ). Using hot biopsy forceps, we grasped the granulation tissue covering the bumper and progressively removed it by alternating between coagulation and cutting modes ( Fig. 1 **b** ). It was not necessary to remove all the granulation tissue covering the entire bumper. Instead, each time granulation tissue was grasped, the hot biopsy forceps were positioned as close as possible to the base of the jejunal tube, which was also the center of the bumper, so that exposure of only a small portion of the central hard structure of the bumper was sufficient to allow proceeding to the next step ( Fig. 1 **c** ). Subsequently, biopsy forceps were introduced through the PEG tube from the external side to grasp a polypectomy snare, which was then drawn through the PEG tube. Following that, the push–pull T technique 4 was employed to pull the buried bumper into the gastric lumen and extract it through the mouth ( Fig. 2 ). A new PEG tube was inserted through the original gastrostomy site, and a replacement jejunal tube was simultaneously placed. Jejunal feeding could commence immediately following the procedure. On the 3rd postoperative day, a follow-up endoscopy demonstrated satisfactory healing at the gastric stoma site ( Fig. 3 ).

**Fig. 1:**
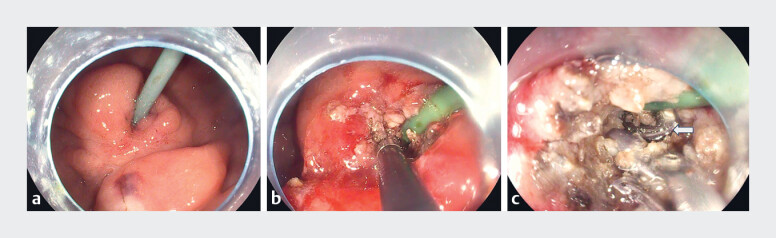
Endoscopic release and replacement of buried bumper in a percutaneous gastrostomy. **a** The jejunal tube enabled identification of the bumper’s center. **b** The granulation tissue over the bumper’s center was removed with hot biopsy forceps. **c** Part of the central hard structure of the bumper (arrow) was exposed.

**Fig. 2:**
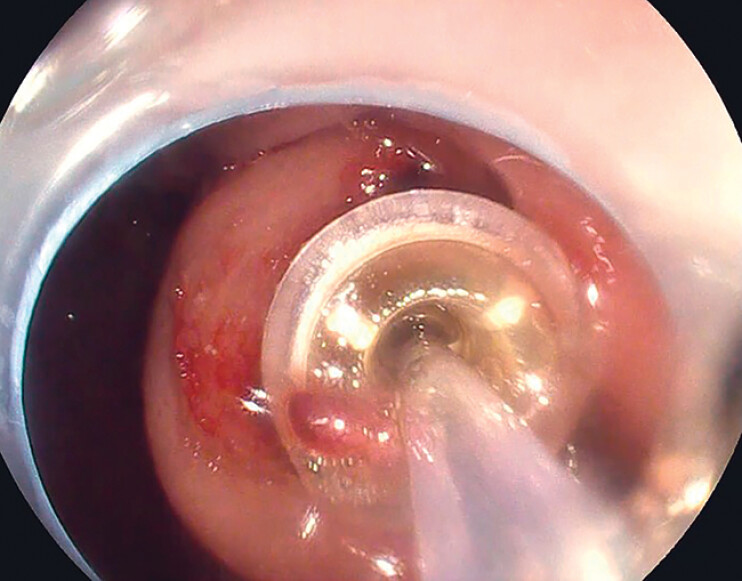
The push–pull T technique was employed to easily release the buried bumper.

**Fig. 3:**
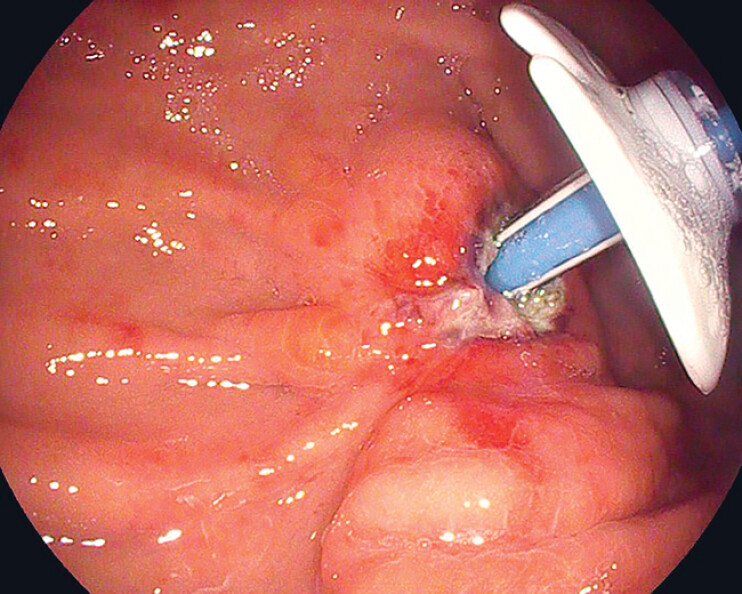
The gastric stoma site showed good healing on the 3rd postoperative day.

This method does not require dedicated devices or complex endoscopic techniques, making it an effective, economical, and safe approach for treating BBS.

Endoscopy_UCTN_Code_TTT_1AO_2AK

## Notes

Endoscopy E-Videos https://eref.thieme.de/e-videos
*E-Videos* is an open access online section of the journal *Endoscopy* , reporting on interesting cases and new techniques in gastroenterological endoscopy. All papers include a high-quality video and are published with a Creative Commons CC-BY license. Endoscopy E-Videos qualify for HINARI discounts and waivers and eligibility is automatically checked during the submission process. We grant 100% waivers to articles whose corresponding authors are based in Group A countries and 50% waivers to those who are based in Group B countries as classified by Research4Life (see: https://www.research4life.org/access/eligibility/ ). This section has its own submission website at https://mc.manuscriptcentral.com/e-videos .
